# An 8-gene machine learning model improves clinical prediction of severe dengue progression

**DOI:** 10.1186/s13073-022-01034-w

**Published:** 2022-03-29

**Authors:** Yiran E. Liu, Sirle Saul, Aditya Manohar Rao, Makeda Lucretia Robinson, Olga Lucia Agudelo Rojas, Ana Maria Sanz, Michelle Verghese, Daniel Solis, Mamdouh Sibai, Chun Hong Huang, Malaya Kumar Sahoo, Rosa Margarita Gelvez, Nathalia Bueno, Maria Isabel Estupiñan Cardenas, Luis Angel Villar Centeno, Elsa Marina Rojas Garrido, Fernando Rosso, Michele Donato, Benjamin A. Pinsky, Shirit Einav, Purvesh Khatri

**Affiliations:** 1 Institute for Immunity, Transplantation and Infection, School of Medicine, Stanford University, CA Stanford, USA; 2 Cancer Biology Graduate Program, School of Medicine, Stanford University, CA Stanford, USA; 3 Division of Infectious Diseases and Geographic Medicine, Department of Medicine, School of Medicine, Stanford University, CA Stanford, USA; 4 Immunology Graduate Program, School of Medicine, Stanford University, CA Stanford, USA; 5 Department of Pathology, School of Medicine, Stanford University, CA Stanford, USA; 6 Clinical Research Center, Fundación Valle del Lili, Cali, Colombia; 7 Centro de Atención y Diagnóstico de Enfermedades Infecciosas (CDI), Bucaramanga, Colombia; 8 Division of Infectious Diseases, Department of Internal Medicine, Fundación Valle del Lili, Cali, Colombia; 9 Center for Biomedical Informatics Research, Department of Medicine, School of Medicine, Stanford University, CA Stanford, USA; 10 Department of Microbiology and Immunology, School of Medicine, Stanford University, CA Stanford, USA

**Keywords:** Dengue, Severe dengue, Prognostic, Biomarkers, Machine learning, Gene signature, Host response

## Abstract

**Background:**

Each year 3–6 million people develop life-threatening severe dengue (SD). Clinical warning signs for SD manifest late in the disease course and are nonspecific, leading to missed cases and excess hospital burden. Better SD prognostics are urgently needed.

**Methods:**

We integrated 11 public datasets profiling the blood transcriptome of 365 dengue patients of all ages and from seven countries, encompassing biological, clinical, and technical heterogeneity. We performed an iterative multi-cohort analysis to identify differentially expressed genes (DEGs) between non-severe patients and SD progressors. Using only these DEGs, we trained an XGBoost machine learning model on public data to predict progression to SD. All model parameters were “locked” prior to validation in an independent, prospectively enrolled cohort of 377 dengue patients in Colombia. We measured expression of the DEGs in whole blood samples collected upon presentation, prior to SD progression. We then compared the accuracy of the locked XGBoost model and clinical warning signs in predicting SD.

**Results:**

We identified eight SD-associated DEGs in the public datasets and built an 8-gene XGBoost model that accurately predicted SD progression in the independent validation cohort with 86.4% (95% CI 68.2–100) sensitivity and 79.7% (95% CI 75.5–83.9) specificity. Given the 5.8% proportion of SD cases in this cohort, the 8-gene model had a positive and negative predictive value (PPV and NPV) of 20.9% (95% CI 16.7–25.6) and 99.0% (95% CI 97.7–100.0), respectively. Compared to clinical warning signs at presentation, which had 77.3% (95% CI 58.3–94.1) sensitivity and 39.7% (95% CI 34.7–44.9) specificity, the 8-gene model led to an 80% reduction in the number needed to predict (NNP) from 25.4 to 5.0. Importantly, the 8-gene model accurately predicted subsequent SD in the first three days post-fever onset and up to three days prior to SD progression.

**Conclusions:**

The 8-gene XGBoost model, trained on heterogeneous public datasets, accurately predicted progression to SD in a large, independent, prospective cohort, including during the early febrile stage when SD prediction remains clinically difficult. The model has potential to be translated to a point-of-care prognostic assay to reduce dengue morbidity and mortality without overwhelming limited healthcare resources.

**Supplementary Information:**

The online version contains supplementary material available at 10.1186/s13073-022-01034-w.

## Background

Dengue virus (DENV) is endemic in over 100 countries, with nearly half the global population at risk for infection [1]. The global incidence of dengue continues to rise, with increasing frequency of localized outbreaks. Among the estimated 60 million people per year who develop acute dengue fever, 5-10% progress within several days to severe dengue (SD), a potentially life-threatening complication characterized by plasma leakage, hemorrhage, shock, and/or organ damage [2–5].

Early identification of SD progressors and timely administration of supportive care are essential to reduce morbidity and mortality. In response to this need, the World Health Organization (WHO) introduced new diagnostic subclassifications in 2009: dengue without warning signs (D), dengue with warning signs (DWS), and severe dengue (SD). Under these criteria, the WHO recommends that dengue patients with any clinical warning signs for SD be admitted to the hospital for close monitoring [6]. However, warning signs are broadly defined and nonspecific [7–9]. As a result, their implementation has substantially increased the number of patients admitted to hospitals, many of whom recover without severe complications [10–13]. Moreover, warning signs may develop late in the disease course, precluding early identification of SD progressors [2, 13]. Therefore, there is an urgent need to develop more accurate prognostic tools to reduce dengue morbidity, mortality, and healthcare burden.

Several such tools have been proposed to date, including host response-based gene signatures [14–17] and machine learning models using clinical features or genomic variants [18–21]. However, none have translated to clinical practice due to insufficient predictive power, lack of generalizability, and/or lack of parsimony [16, 22, 23]. We previously identified a 20-gene set associated with SD [24], but its performance in a large, prospective cohort remains unknown. Furthermore, an accurate signature based on fewer genes would be more technically feasible for assay development and cost-effective in limited-resource settings.

Here, we trained an 8-gene machine learning model to predict progression to SD and validated it in an independent prospective cohort. We improved upon existing work in four important ways: (1) we integrated more publicly available data, (2) we applied a modified iterative multi-cohort analysis method to identify genes robustly associated with progression to SD, (3) we trained a machine learning model using signature genes to learn complex underlying patterns within the data, and (4) we validated the model, with locked parameters, in a large, independent, prospectively-enrolled cohort of 377 dengue patients in Colombia. In this prospective cohort, we show the improved generalizability of the 8-gene model compared to the prior 20-gene set. Additionally, we demonstrate the increased prognostic power of the 8-gene model relative to clinical warning signs, indicating the model’s potential utility in guiding dengue patient triage and management.

## Methods

### Curation of public dengue datasets

We searched the NCBI Gene Expression Omnibus (GEO) repository through August 1, 2019, using the query “dengue” for datasets profiling blood gene expression by array or high-throughput sequencing in human dengue patients with varying infection outcomes. We excluded studies that only examined uncomplicated dengue patients or that did not report sample-level information on disease severity. We further excluded cell culture studies, studies on steroid-treated patients, and studies where over half of the genes profiled had sparse expression data (missing values for ≥15% of samples). The resulting 11 datasets were biologically, clinically, and technically heterogeneous, representing seven different countries, patients of all ages, different sample types (whole blood and PBMCs), and distinct technologies for gene expression profiling (Table 1) [14, 15, 25–32]. We classified patients with uncomplicated dengue fever (DF) as “non-severe” and patients with dengue hemorrhagic fever (DHF) or dengue shock syndrome (DSS) as “SD progressors.” We used samples from a total of 365 patients (199 non-severe, 166 SD progressors) for multi-cohort analysis. Additional pre-processing details are described in Additional file 1: Supplementary methods.

**Table 1:** Publicly available datasets used for discovery of the 8-gene set and training of the 8-gene XGBoost model. Healthy controls, convalescent patients, and patients with other febrile illnesses were removed. Longitudinal samples were excluded for gene set discovery and model training but included for temporal gene expression analysis (included in “Total samples used”). *WB*, whole blood; *PBMC*, peripheral blood mononuclear cells

Dataset	Platform	Year	Reference	Country	Age	Tissue	Samples used in discovery	Total samples used
GSE40628	GPL16021 (Lymphochip)	2007	Simmons CP [25]	Vietnam	Adults	WB	14	14
GSE18090	GPL570 (Affymetrix)	2009	Nascimento EJ [26]	Brazil	Adults	PBMC	18	18
GSE13052	GPL2700 (Illumina)	2009	Long HT [27]	Vietnam	Children	WB	18	18
GSE25001	GPL6104 (Illumina)	2010	Hoang LT [28]	Vietnam	Children/adults	WB	96	168
GSE17924	GPL4133 (Agilent)	2010	Devignot S [29]	Cambodia	Children	WB	48	48
GSE38246	GPL15615 (Illumina)	2012	Popper SJ [30]	Nicaragua	Children	PBMC	41	102
GSE43777	GPL201 (Affymetrix)	2013	Sun P [31]	Venezuela	Children/adults	PBMC	26	112
GSE43777	GPL570 (Affymetrix)	2013	Sun P [31]	Venezuela	Children/adults	PBMC	20	74
GSE51808	GPL13158 (Affymetrix)	2014	Kwissa M [32]	Thailand	Adults	WB	28	28
GSE94892	GPL16791 (Illumina)	2017	Banerjee A [14]	India	Children/adults	PBMC	31	31
GSE100299	GPL17586 (Affymetrix)	2017	Simon-Lorière E [15]	Cambodia	Children	PBMC	25	25
**Total**							365	638

### Multi-cohort analysis and gene set discovery

We compared gene expression between non-severe patients and SD progressors by applying Monte Carlo cross-validation at the dataset level to our previously described multi-cohort analysis framework, MetaIntegrator (https://cran.r-project.org/web/packages/MetaIntegrator/) [23], which has enabled identification of several host response-based gene signatures that have been validated independently by our group and others [33–35]. In each iteration, we randomly selected seven datasets for “training” and performed multi-cohort analysis using MetaIntegrator. We applied significance thresholds ∣effect size (ES) ∣  ≥ 0.4 and false discovery rate ≤ 10% to identify differentially expressed genes (DEGs). We then performed multi-cohort analysis on the remaining four “validation” datasets to identify the genes from training that also passed the significance threshold ∣effect size (ES) ∣  ≥ 0.25 in validation. After 100 iterations, we derived a set of 25 DEGs that were significant in both training and validation in greater than 50% of the iterations. From this gene set, we performed a greedy forward search, as implemented in MetaIntegrator, to identify the most predictive gene set. Briefly, we added genes one-by-one to optimize area under the receiver operating characteristic (ROC) curve (AUC) across all 11 datasets (weighted by sample size in each dataset) using the difference-of-geometric-means score, computed by subtracting the geometric mean of the downregulated genes from the geometric mean of the upregulated genes. This resulted in an 8-gene set with three upregulated genes and five downregulated genes.

### Longitudinal analysis in public datasets

For longitudinal analysis of the eight genes in the public datasets, we used the seven datasets that reported day post-fever onset at the sample level (GSE13052, GSE17924, GSE18090, GSE25001, GSE38246, GSE43777_GPL201, GSE43777_GPL570). We used a total of 539 samples (342 from non-severe patients, 197 from SD progressors), including those from multi-cohort analysis and additional longitudinal samples. We calculated and analyzed standardized expression values for the eight genes across all seven datasets over the disease course (see Additional file 1: Supplementary methods). We performed smoothing using local weighted regression (LOESS) [36].

### Model generation

#### Data pre-processing

We combined the public datasets and generated a reduced gene expression matrix consisting of the 25 DEGs from multi-cohort analysis and the prior SD-associated 20-gene set [24] (43 unique genes in total). We excluded two genes that were missing from five datasets, and two datasets (GSE43777_GPL201 and GSE40628) that were missing over a third of the genes. We imputed the remaining missing values using *missForest* [37] and adjusted for batch effects using *ComBat* [38].

#### Linear models

We built two linear models based on the eight genes to classify non-severe patients and SD progressors. The first model used the difference-of-geometric-means score, computed as described above. The second was a logistic regression model with the eight genes as predictors.

#### Age-dependent performance

Most public datasets did not include sample-level age information. We therefore compared model performance in datasets that profiled different age groups (four datasets profiled children only, three profiled adults only, and four profiled both children and adults).

#### XGBoost model

We trained an XGBoost gradient-boosted tree model [39] using only the eight genes as features. We held out one dataset (GSE100299) from hyperparameter tuning to check for overfitting in silico prior to testing the model in an independent, prospective cohort from Colombia (see below). On the remaining eight datasets consisting of 300 samples (146 severe, 154 non-severe), we performed XGBoost hyperparameter tuning using *Caret* [40] with leave-one-dataset-out cross-validation, which is shown to be less biased than *k*-fold cross-validation [41]. We “locked” the model using the set of parameters that maximized AUC in cross-validation and in the held-out dataset. The final locked model had the following parameters and hyperparameters: nrounds = 40, max_depth = 2, min_child_weight = 4, subsample = 0.9, colsample_bytree = 0.5, eta = 0.2, gamma = 0.9, nthread = 1, and scale_pos_weight = 1.5. We assessed feature importance using the “gain” metric as calculated by XGBoost, which represents the relative contribution of each of the eight genes to model accuracy.

### Model performance metrics

#### Summary ROC curves

The summary ROC curve represents a weighted average of multiple independent ROC curves and was calculated as follows. True positive rate (TPR) values for each curve were approximated using linear interpolation, and the summary ROC curve was calculated using the mean of the TPR values for each curve, weighted based on sample size. A weighted standard deviation was also calculated for each TPR and is depicted by the shaded gray area around the summary ROC curve. The area under the summary ROC curve (summary AUC) was calculated using the trapezoid rule. Finally, the 95% confidence interval (CI) for the summary AUC was calculated using the pooled standard error of the individual AUCs.

#### Number needed to predict (NNP)

NNP, like number needed to treat (NNT), is a metric of diagnostic or prognostic accuracy that provides a more intuitive benchmark for clinicians than other measures of accuracy [42–44]. Here, NNP is defined as the number of dengue patients who need to be examined in order to accurately predict that one patient will progress to SD. NNP is equal to the inverse of the predictive summary index (PSI), or 1/(PPV + NPV – 1).

#### CIs for performance metrics

We computed 95% CIs for individual AUCs using the DeLong method as implemented in the *pROC* package in R [45, 46]. We computed 95% CIs for sensitivity, specificity, positive predictive value (PPV), negative predictive value (NPV), and NNP using stratified bootstrapping, with the proportion of SD cases held constant across all 10,000 bootstrap samples. We used the same cut-off point for 8-gene model predicted probabilities across all bootstrap samples, set at the Youden threshold of the original dataset. For likelihood ratios, we used the alternative bootstrapping approach described in Marill et al. to obtain appropriate CIs when sample sensitivity was 100% [47].

### Benchmarking against published gene signatures

We identified 12 studies that described sets of DEGs in dengue patients (Additional file 2: Table S1). Of these, we excluded two studies that did not describe severity-associated DEGs, two that described hundreds or thousands of DEGs which would not be suitable for translation to a point-of-care test, and five studies that provided a list of top severity-associated DEGs without any classification model for severity prediction. We additionally excluded the study by Nikolayeva et al. [17] due to insufficient methodological details and lack of access to their training data with which to reproduce their model. The remaining two studies (Nascimento et al. [26] and Robinson et al. [24]) described reproducible classification models using parsimonious gene signatures to predict progression to SD. We therefore assessed the performance of both models in unseen public datasets, excluding those that had been used for training each respective model.

### Independent prospective Colombia cohort

#### Ethics Statement

All work with human subjects was approved by the Stanford University Administrative Panel on Human Subjects in Medical Research (protocols #35460 and #50513) and the ethics committees in biomedical research of the Fundación Valle del Lili (FVL, Cali, Colombia) and Centro de Atención y Diagnóstico de Enfermedades Infecciosas (CDI, Bucaramanga, Colombia). All subjects or their parents or legal guardians provided written informed consent, and subjects between 2 and 17 years of age and older provided assent. Subjects were not involved in previous procedures and were all test-naïve.

#### Study population and sample collection

The independent, prospective Colombia cohort consisted of 377 individuals presenting to the emergency room or clinics of FVL or CDI between March 2016 and Jan 2020. Enrollment criteria consisted of (i) age equal to or greater than 2 years; (ii) presentation with an acute febrile illness of less than seven days associated with one or more of the following symptoms or signs: headache, rash, arthralgia, myalgia, retroorbital pain, abdominal pain, positive tourniquet test, petechiae, and bleeding; and (iiia) a positive dengue IgM antibody and/or NS1 antigen by the SD BIOLINE Dengue Duo combo device (Standard Diagnostic Inc., Korea) test [48] or (iiib) clinical presentation highly consistent with dengue and subsequent confirmation of diagnosis via rRT-qPCR at Stanford (see Additional file 1: Supplementary methods). Patients were classified by infectious diseases specialists, both upon presentation and following the end of the disease course, as having dengue (D), dengue with warning signs (DWS), or severe dengue (SD) according to 2009 WHO criteria [49, 50] (Table 2). Venous blood samples were collected upon enrollment on the first day of presentation. 2.5 ml of whole blood were collected in Paxgene tubes (PreAnalytiX) and stored at − 80 °C. Serum samples were obtained for additional assays. Sample transport, reception, and processing were strictly controlled using personal data assistants (PDAs) with barcode scanners.

**Table 2:** Summary of demographic information and clinical parameters of the independent prospective Colombia cohort. For days from sample to severe dengue (SD) onset, “0” indicates patients whose sample was collected on the day of (at least several hours prior to) the appearance of SD manifestations. *WS*, warning signs; *NS1 Ag*, nonstructural protein 1 antigen; *DENV*, dengue virus

		Dengue (***N***=93)	Dengue with WS (***N***=262)	Severe dengue (***N***=22)
**Age**	Adult	39	86	13
	Child (<17 years)	54	176	9
**Gender**	Male	49	137	6
	Female	44	125	16
**Total**		93	262	22
**First sample day**	Mean (range)	5.0 (1–10)	5.2 (0–10)	4.8 (0–7)
**Days from sample to SD onset**	Median (range)	-	-	− 1 (− 3, 0)
**Dengue diagnostics**	Positive NS1 Ag	60	215	17
	Positive DENV IgM	53	149	9
**Dengue exposure**	Primary	29	59	5
	Secondary	59	186	15
	Undetermined	5	17	2
**Dengue serotype**	DENV-1	40	135	10
	DENV-2	-	3	-
	DENV-3	1	3	3
	DENV-4	2	3	1
	DENV co-infected	-	1	-
	Unknown	50	117	8

Of 399 patients who consented, four patients were excluded following confirmatory rRT-PCR analysis at Stanford (one false positive, three with Zika virus co-infection) (Additional file 3: Fig. S1). Another five patients were excluded who already displayed SD manifestations upon presentation. RNA was extracted from the remaining 390 blood samples, of which nine were excluded due to low RNA concentrations and another four excluded for other technical reasons. Altogether, 377 blood samples were analyzed for mRNA expression of the signature genes using NanoString.

For 154 of 377 patients who were managed in the outpatient setting, follow-up was conducted daily via phone, during which patients were provided information about the clinical warning signs and asked about their appearance, until full recovery when final diagnoses were determined. For all patients, final diagnoses were blindly re-classified by infectious diseases specialists according to the 1997 WHO criteria into DF, DHF, and/or DSS [51]. Organ damage was defined according to standard clinical endpoints for DENV infection [52]. Demographics and clinical information were collected at the time of presentation. The first day of fever (fever day 0) was defined by the patients or their relatives. Symptoms, warning signs, and laboratory parameters (including complete blood count, chemistry, and liver function test results) were documented by healthcare professionals (Additional file 4: Table S2). Serotype and prior DENV exposure were determined through rRT-PCR and serological assays at Stanford (see Additional file 1: Supplementary methods).

#### RNA extraction and gene expression measurement

RNA was extracted from PAXgene RNA tubes using the PAXgene Blood RNA Kit IVD (Qiagen) according to the manufacturer’s protocol and analyzed for RNA quality using the Agilent bioanalyzer QC analysis. RNA was concentrated using RNA Clean & Concentrator-5 kit (Zymo Research) according to the manufacturer’s protocol. Gene expression was measured using NanoString nCounter technology. Each NanoString expression profiling reaction consisted of 300 ng of total RNA per 15 uL sample, hybridized for 16 h at 65° C per the manufacturer’s instructions. nCounter SPRINT standard protocol was used to generate mRNA counts. A quality control check was performed according to the manufacturer’s guidelines for assessing imaging quality, binding density, positive control linearity, and the limit of detection. Raw mRNA counts were normalized to *EIF6* and *ILF2* as housekeeping genes.

#### Power analysis

We calculated statistical power to detect a range of AUCs based on SD prevalence in the Colombia cohort using the method of Obuchowski and McClish [53], as implemented in the *pROC* R package [45].

#### Association of model predictions with clinical features

We examined the association between 8-gene model predicted probabilities and various clinical features using the Wilcoxon rank-sum test and Pearson correlation coefficient for categorical or continuous variables, respectively.

### Model calibration

We calibrated our model using the Platt scaling method [54]. We resampled control samples in the training data, with replacement, to achieve the same prior probability of SD as observed in the independent cohort (5.8%). We applied the Platt scaling method to this dataset of 2702 (resampled) controls and 157 cases, fitting a logistic regression model on the 8-gene model predicted probabilities. We then applied this logistic regression model to transform the 8-gene model predicted probabilities for the independent cohort.

### Generalizability of the 8-gene model to other viral infections

We identified four public datasets, preprocessed as described previously [55], consisting of samples from 336 patients with SARS-CoV-2, influenza virus, respiratory syncytial virus (RSV), or chikungunya virus infection (Additional file 2: Table S3) [56–60]. Patients were of all ages, enrolled across six different countries, profiled using microarray or RNA sequencing technologies, with mild/moderate (*N*=166) or severe (*N*=170) infection. We excluded healthy controls and patients who were asymptomatic or convalescent. We calculated 8-gene signature scores based on the difference of geometric means and examined the performance of the 8-gene signature in classifying patients with mild/moderate or severe infection.

## Results

### Identification of an 8-gene set associated with progression to severe dengue

**Fig. 1:**
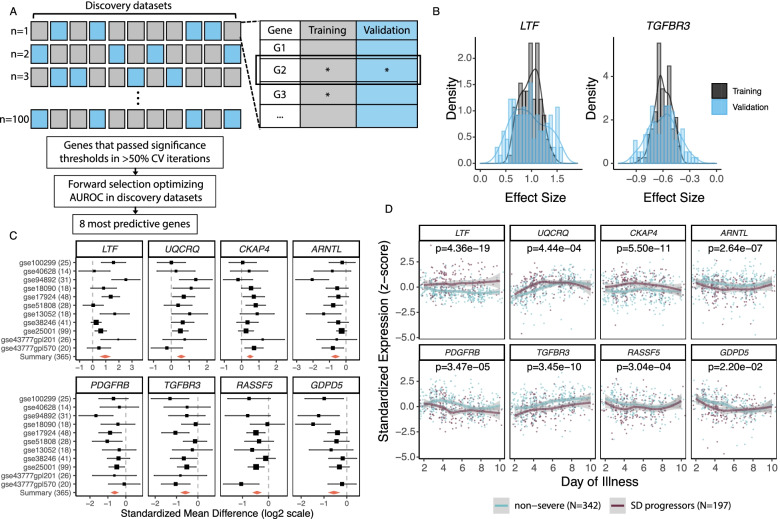
Multi-cohort analysis identifies eight genes robustly associated with progression to SD. **A** Schematic of multi-cohort analysis method with Monte Carlo sampling at the dataset level. In each of 100 cross-validation (CV) iterations, we randomly selected seven datasets for “training” (gray), identified differentially expressed genes (DEGs) using MetaIntegrator, and examined them in the remaining four “validation” (blue) datasets. DEGs that passed significance thresholds (as denoted by asterisks) in both training and validation were considered significant for that iteration. We then did a greedy forward search on DEGs significant in greater than 50% of all iterations and identified the eight most predictive DEGs. **B** Representative plots of the distribution of effect size (log2) in training (gray) and validation (blue) across the 100 iterations for over-expressed (*LTF*) and under-expressed (*TGFBR3*) genes that passed significance thresholds in >50% of iterations. Regardless of the combination of datasets in training or validation, the distribution of effect sizes for all 25 genes did not contain 0. **C** Forest plot of the effect size of the eight genes in each discovery dataset. Two genes (*RASSF5* and *GDPD5*) were not measured in every dataset. The black lines indicate the 95% confidence interval (CI) of the effect size for a given gene in a given dataset, and the size of the black box is proportional to the sample size of each dataset. The summary effect size of each gene across all datasets is indicated by the red diamond; the width of the diamond indicates the 95% CI. **D** Standardized expression of each of the eight genes over the disease course (days post-symptom onset) in patients who remained non-severe (blue) or progressed to SD (purple). Seven discovery datasets that reported day of sample collection were included in longitudinal analysis. Lines represent the local regression (LOESS) curve fit for non-severe patients and SD progressors. Gray bands represent the 95% CI

We identified 11 publicly available datasets that profiled the blood transcriptome in 365 dengue patients, of which 199 remained non-severe and 166 progressed to SD (Table 1) [14, 15, 25–32]. These datasets collectively encompassed biological, clinical, and technical heterogeneity. To identify DEGs robustly associated with progression to SD across all 11 public datasets, we developed a novel method for multi-cohort analysis, using MetaIntegrator [23] with iterative Monte-Carlo sampling at the dataset level (Methods, Fig. 1A). We found 25 significant DEGs with consistent effect size across all iterations (Fig. 1B). Among these 25 DEGs, our previously described greedy forward search [61] selected eight, of which three were over-expressed (*LTF*, *UQCRQ*, *CKAP4*) and five under-expressed (*ARNTL*, *PDGFRB*, *TGFBR3*, *RASSF5*, *GDPD5*) in SD progressors (Additional file 2: Table S4, Fig. 1C). While *LTF, CKAP4*, and *TGFBR3* were differentially expressed between SD progressors and non-progressors throughout the disease course, the remaining five genes exhibited differential expression in the first 3–6 days post-fever onset and converged for the remainder of the disease course (Fig. 1D).

Collectively, these results show that there is an early blood transcriptional response to DENV infection that is robustly associated, during the acute febrile stage, with subsequent progression to SD across heterogeneous patient populations.

### Model generation to predict severe dengue progression in existing cohorts

To build a generalizable model to predict SD progression, we first examined age as a confounding variable, as several studies have described differences in dengue presentation and severity by age [7, 62–65]. Indeed, linear classifiers exhibited age-dependent performance, with area under the receiver operating characteristic curves (AUCs) ≥ 0.85 in datasets profiling children and AUCs ≤ 0.7 in datasets profiling adults (Additional file 3: Fig. S2). We could not include age as a variable due to lack of sample-level age information in many public datasets. Therefore, we turned to a non-linear classifier that could better learn the latent, complex relationships among age, gene expression, and dengue severity.

**Fig. 2:**
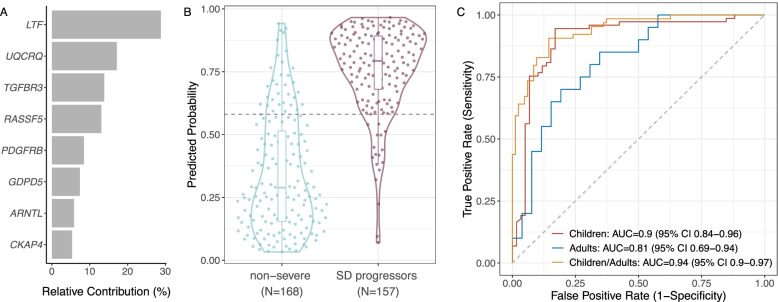
The 8-gene XGBoost-based model predicts progression to SD in public datasets. **A** Relative contribution of each of the eight genes to the XGBoost model. **B** Violin plot of predicted probabilities of progression to SD for samples across all public datasets. The dotted horizontal line indicates the Youden optimal threshold for the public datasets. **C** ROC curves of the 8-gene model predictions for distinguishing non-severe patients from SD progressors in datasets profiling children (red), adults (blue), or both children and adults (orange). The DeLong test p-value for children vs. adults is 0.205

Utilizing the eight genes as features, we trained a machine learning model with XGBoost [39] on public data. The model parameters were tuned and “locked” prior to validation in an independent, prospective cohort from Colombia (see below). The model predictions were driven by four genes, *LTF*, *UQCRQ*, *TGFBR3*, and *RASSF5*, which together had a relative contribution to model accuracy of 72.9% (Fig. 2A). In the public datasets, the 8-gene XGBoost model classified non-severe patients and SD progressors with a summary AUC = 0.891 (95% CI 0.706–1) (Additional file 3: Fig. S3A–B). At the Youden threshold [66], the model had 89.2% (95% CI 84.1–93.8) sensitivity and 81% (95% CI 75.0–86.7) specificity (Fig. 2B). Although the 8-gene model had a higher AUC in children (< 18 years) than adults, this difference was statistically insignificant (DeLong test p-value=0.205) (Fig. 2C, Additional file 3: Fig. S3C). Together, these results suggest that our 8-gene XGBoost model has improved generalizability compared to linear classifiers.

To benchmark performance of the 8-gene XGBoost model, we evaluated two previously published gene signatures for SD that provided sufficient information to be reproduced (Additional file 2: Table S1). When applied to public datasets not used for model training, a 2-gene model by Nascimento et al. performed poorly, but our previously described 20-gene set generalized well to unseen data (Additional file 3: Fig. S4). We therefore assessed the 20-gene set alongside the 8-gene XGBoost model in the prospective cohort as described below.

### Independent validation and comparison to warning signs in a prospective cohort of dengue patients

**Fig. 3:**
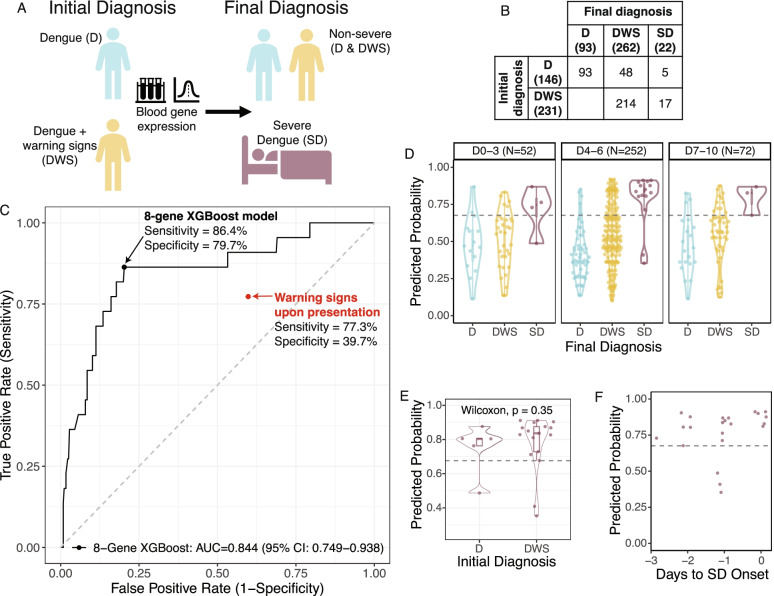
The locked 8-gene XGBoost model predicts progression to SD in an independent prospective dengue cohort. **A** Description of independent Colombia cohort. Blood samples were collected upon presentation from dengue patients presenting with or without warning signs. **B** Confusion matrix depicting the number of patients with an initial diagnosis of D or DWS upon presentation and final diagnosis of D, DWS, or SD. **C** ROC curve of the locked 8-gene XGBoost model in predicting progression to SD in the independent cohort. The black point indicates the sensitivity and specificity of the 8-gene model at the Youden threshold in the independent cohort. The red point indicates the sensitivity and specificity of clinical warning signs in predicting progression to SD in the independent cohort. **D** 8-gene model predictions on samples collected throughout the disease course, on days 0–3, 4–6, or 7–10 post-fever onset. **E** Violin plot of the predicted probabilities of progression to SD for SD progressors in the independent cohort who initially presented with or without warning signs. **F** Predicted probabilities using the 8-gene model for the 22 patients in the independent Colombia cohort who progressed to SD, by days from sample collection to the appearance of severe manifestations (“Days to SD Onset”). “0” indicates patients whose sample was collected on the day of—but at least several hours prior to—the appearance of SD manifestations. The dotted horizontal line indicates the Youden threshold in the Colombia cohort

To independently validate our locked 8-gene XGBoost model, we prospectively enrolled 377 patients with DENV infection in Cali and Bucaramanga, Colombia (Table 2, Additional file 3: Fig. S1, Additional file 4: Table S2). Patients presenting with SD were excluded. We collected whole blood samples at presentation (i.e., prior to progression to SD) and followed patients throughout the course of infection (Fig. 3A). Upon presentation, 231 patients had warning signs (DWS), and 146 did not (D). By the end of the disease course, 22 patients had progressed to SD, defined as one or more of the following: (i) plasma leakage that may lead to shock and/or fluid accumulation, with or without respiratory distress, and/or (ii) severe bleeding, and/or (iii) severe organ impairment [6]. With the proportion of SD cases observed here (5.8%), we had >90% statistical power to detect AUC > 0.70 (Additional file 2: Table S5). The proportion of SD cases was higher in adults (9.4%) than in children (4%).

Of the 231 patients who initially presented with warning signs, 17 (7.4%) progressed to SD, and 214 (92.6%) remained non-severe (Fig. 3B). Importantly, five of 22 (22.7%) SD progressors did not initially present with warning signs. Hence, clinical warning signs at presentation had 77.3% (95% CI 58.3–94.1) sensitivity and 39.7% (95% CI 34.7–44.9) specificity for predicting progression to SD (Fig. 3C), corresponding to positive and negative likelihood ratios of 1.3 (95% CI 0.9–1.6) and 0.6 (95% CI 0.2–1.1), respectively (Table 3). In adults, warning signs were an especially poor predictor of SD, with sensitivity and specificity of 66.7% and 45.2%, respectively (Additional file 2: Table S6). In children, warning signs were more sensitive (90.0%) but less specific (37.1%). Altogether, clinical warning signs upon presentation had a PPV of 7.4% (95% CI 4.3–10.9) and NPV of 96.6% (95% CI 93.3–99.3), resulting in an NNP of 25.4 patients (Table 3). Overall, warning signs upon presentation were a poor predictor of progression to SD.

**Table 3:** Performance of the 8-gene XGBoost model and clinical warning signs in the independent cohort. 95% confidence intervals (CIs) from bootstrapping are shown in parentheses for each metric. For the NNP of warning signs, the lower CI bound is omitted as the 95% CI contained negative values due to the sum of PPV and NPV being less than 1 (indicating no gain in certainty according to the Predictive Summary Index). *LR+*, positive likelihood ratio; *LR−*, negative likelihood ratio; *PPV*, positive predictive value; *NPV*, negative predictive value; *NNP*, number needed to predict

	Sensitivity %	Specificity %	LR+	LR−	PPV %	NPV %	NNP
**8-gene XGBoost model**	86.4 (68.2–100.0)	79.7 (75.5–83.9)	4.3 (3.2–5.5)	0.2 (0.01–0.4)	20.9 (16.7–25.6)	99.0 (97.7–100.0)	5.0 (4.0–6.8)
**Warning signs**	77.3 (58.3–94.1)	39.7 (34.7–44.9)	1.3 (0.9–1.6)	0.6 (0.2–1.1)	7.4 (4.3–10.9)	96.6 (93.3–99.3)	25.4 (NA–185.6)

Next, we applied the 8-gene XGBoost model to whole blood samples obtained upon presentation, prior to progression to SD. In this independent prospective cohort where gene expression was measured on a different platform (NanoString), the locked 8-gene XGBoost model predicted subsequent progression to SD with an AUC of 0.844 (95% CI 0.749–0.938) (Fig. 3C). At the Youden threshold, the 8-gene XGBoost model had a sensitivity of 86.4% (95% CI 68.2–100.0), specificity of 79.7% (95% CI 75.5–83.9), and positive and negative likelihood ratios of 4.3 (95% CI 3.2–5.5) and 0.2 (95% CI 0.01–0.4), respectively (Table 3). Compared to warning signs, the 8-gene model had a substantially higher PPV of 20.9% (95% CI 16.7–25.6) and NPV of 99.0% (95% CI 97.7–100.0). This resulted in an area under the precision-recall curve (AUPRC) of 0.280 (Additional file 3: Fig. S5A) and an 80% reduction in the NNP to 5.0 (95% CI 4.0–6.8).

The 8-gene model exhibited statistically insignificant differences by age (DeLong *p* = 0.19), outperforming clinical warning signs for both age groups with AUCs of 0.751 (95% CI 0.601–0.901) and 0.889 (95% CI 0.747–1) in adults and children, respectively (Additional file 2: Table S6, Additional file 3: Fig. S5B). In contrast, the prior 20-gene set performed significantly worse in adults (AUC = 0.665, 95% CI 0.501–0.830) than in children (AUC = 0.936, 95% CI 0.881–0.991; DeLong *p* = 0.0026). Thus, the 8-gene XGBoost model improved prediction for both age groups compared to clinical warning signs and was more generalizable than the 20-gene set.

As the early prediction of SD is of utmost importance given the rapid nature of progression following defervescence, we next examined the performance of the 8-gene model by time in the disease course. The 8-gene model was predictive of subsequent SD on samples collected throughout the disease course, including those from the first three days of acute fever (Fig. 3D). For most patients, this is prior to the manifestation of clinical warning signs [1]; indeed, the 8-gene model accurately predicted subsequent SD even for patients who, at the time of sample collection, did not present with warning signs (Fig. 3E). Furthermore, for SD patients, the 8-gene model predictions were accurate up to three days before progression to SD (Fig. 3F).

Finally, we analyzed the performance of the 8-gene model against the 1997 WHO criteria used in the public datasets (DF, DHF, DSS) (Additional file 4: Table S2) [51]. The 8-gene model performed comparably with the 1997 criteria (AUC = 0.842, 95% CI 0.716–0.968) (Additional file 3: Fig. S5C–D). Notably, although the model was not trained to classify patients with organ damage—a severe complication included in 2009, but not 1997, criteria—seven of eight patients in our independent cohort who developed organ damage without severe hemorrhage or shock were accurately predicted by the 8-gene model as SD progressors.

Collectively, these results demonstrate the early prognostic power and generalizability of the 8-gene model applied to samples collected at presentation in a large, independent, prospectively enrolled cohort. Of note, due to differences in class balance between the public datasets used for training and the independent validation cohort, our model probabilities were not calibrated; however, calibrated probabilities generated through monotonic transformation did not change any of our conclusions (Fig. S6).

### Association of clinical features with 8-gene model predicted probabilities

We next examined the relationship between the 8-gene model predictions and relevant clinical features. The 8-gene model predicted probabilities were significantly higher in patients with prior exposure to DENV than those without; nonetheless, the model accurately distinguished SD progressors with primary or secondary infection (Additional file 3: Fig. S7A). Additionally, the 8-gene model predictions were positively associated with fluid accumulation but not with vomiting, hemorrhage, abdominal pain, or hepatomegaly (Additional file 3: Fig. S7B–F). The 8-gene model predictions were significantly, moderately positively correlated with peak alanine transaminase (ALT) and aspartate transaminase (AST) and moderately negatively correlated with platelet nadir (Fig. S7G–I).

### Generalizability of the 8-gene model to other viral infections

We have previously described a conserved host response to a broad range of viral infections that are associated with disease severity [34, 55]. We therefore assessed whether the 8-gene set may also predict severity in other viral infections. We identified four independent cohorts consisting of 336 patients (166 mild/moderate and 170 severe) infected with SARS-CoV-2, chikungunya, influenza, or respiratory syncytial virus (RSV) (Additional file 2: Table S3) [56–60]. The 8-gene signature distinguished mild/moderate infection from severe infection for SARS-CoV-2, influenza, and RSV, but not chikungunya (Fig. S8). These preliminary results suggest that the 8-gene signature may have some discriminatory power in other viral infections.

## Discussion

Clinical warning signs, designed to guide dengue patient triage, have poor sensitivity and specificity for predicting progression to SD [7–13], as reflected in the present study. An accurate prognostic assay for SD could improve early detection of SD and reduce healthcare burden. Here, we leveraged the substantial biological, clinical, and technical heterogeneity in publicly available dengue datasets and identified an 8-gene set associated with SD, from which we built an XGBoost-based machine learning model to predict progression to SD. We validated the locked 8-gene model in a large, independent, prospective cohort. Applied to blood samples collected prior to SD, the 8-gene model accurately predicted progression to SD, including in the early febrile stage. Compared to clinical warning signs, which were pervasive, nonspecific, and insufficiently sensitive, the 8-gene model reduced the NNP by 80%, demonstrating potential for translation to a point-of-care assay for SD prediction.

Though the eight genes were robustly associated with SD across heterogeneous patient cohorts, their biological roles in the DENV host response are largely unknown, except for lactotransferrin (*LTF*), a non-specific antimicrobial peptide upregulated in SD patients as well as in patients with severe sepsis [27, 67–69]. While the other seven genes have not been specifically studied in dengue, they are known to be involved in pathways that have been implicated in DENV or other viral infections. For instance, TGFβ signaling has been associated with dengue severity [70], and the RAS pathway may be a target of miRNAs expressed in DHF [71]. Interestingly, six of the eight genes were differentially expressed in SD progressors early in the disease course (days 2–6), indicating that they are biomarkers of a defective early host response rather than signs of ongoing SD pathogenesis. Such temporal dynamics likely contribute to the predictive nature of the 8-gene set. Moreover, they suggest that early therapeutic interventions for high-risk patients may be effective in reducing or preventing subsequent morbidity and mortality.

The 8-gene XGBoost model offers two major improvements to dengue patient triage: generalizability and early prognostic power. We found highly age-dependent performance of clinical warning signs and the prior 20-gene set in the independent Colombia cohort. While linear models using the 8-gene set also exhibited differences by age in the public datasets, our 8-gene XGBoost model mitigated these differences and outperformed clinical warning signs in both children and adults in the Colombia cohort. Moreover, despite being trained on datasets that used the 1997 WHO criteria [51], the 8-gene model accurately predicted SD in patients with various clinical manifestations in the Colombia cohort, including those presenting with organ damage in the absence of DHF or DSS. Additionally, the 8-gene XGBoost model predicted SD progression for patients whose samples were collected in the first three days of fever, prior to the appearance of warning signs, and up to three days before onset of SD. In this early febrile stage of illness, it remains clinically difficult to accurately predict subsequent outcomes [72]. Therefore, the 8-gene model has potential to improve SD prediction, particularly in the early stages of disease.

The generalizability of the 8-gene XGBoost model reflects the robustness of our methods for gene selection and model training. Regarding gene selection, the multi-cohort analysis framework with Monte-Carlo sampling used here provides important advantages over approaches that merge independent datasets through batch correction. First, attempts to eliminate technical heterogeneity using batch correction may inadvertently reduce the meaningful biological and clinical heterogeneity across independent patient cohorts [22, 73, 74]. Preservation of this biological and clinical heterogeneity is necessary for the discovery of a gene signature that generalizes to the real-world dengue patient population [22]. Second, data-merging approaches preclude estimation of intra- and inter-dataset variability of each gene, which can be useful for gene selection. In contrast, our multi-cohort analysis approach uses these features as weights when computing gene-wise effect size and standard error, and additionally estimates between-dataset heterogeneity for each gene, which can be utilized for gene selection [23]. Third, unlike an approach that merges all datasets and analyzes them simultaneously, the iterative Monte-Carlo sampling approach ensures that datasets with large sample sizes do not have undue influence on gene selection. Moreover, it requires that genes are robustly associated with progression to SD, regardless of the combination of datasets used for in silico training and validation.

Regarding model training, we took several steps to avoid overfitting. We avoided the curse of dimensionality [75] by restricting the model to the pre-selected genes from the multi-cohort analysis. Next, we performed leave-one-dataset-out cross-validation for model training and hyperparameter tuning, preventing the information leakage that occurs with *k*-fold cross-validation [41]. Finally, we held out an additional public dataset from model training and hyperparameter tuning for an in silico evaluation of overfitting prior to locking the model for independent validation in the Colombia cohort.

While the 8-gene set was discovered in dengue datasets, it also distinguished severe and non-severe patients with other viral infections with reasonably high accuracy. However, it had lower accuracy in other viral infections than other host response-based signatures [55] and did not generalize to chikungunya patients. While further validation is needed in other cohorts and viral infections, these preliminary findings suggest that the 8-gene set is comprised of elements of the conserved host response to viral infection [34, 55] as well as elements more specific to DENV infection.

Future work should focus on evaluating clinical relevance and implementation of the 8-gene model. In the present study, model predictions were associated with some, but not all, clinical warning signs and SD risk factors, suggesting it may provide useful information beyond existing clinical measures. Assessing the combined predictive power of the 8-gene model with various demographic and clinical features should be a priority area for future research, as it may enable an enhanced prognostic algorithm that accounts for age and other factors to further improve accuracy. This work would also inform the design of clinical trials to determine the optimal stage(s) for implementation of such an assay in the clinical workflow: whether as an additional warning sign for SD, a rule-out test for non-severe dengue patients, and/or an additional parameter to further partition DWS patients when allocation of limited resources is needed.

Our study has several limitations. First, it is possible that hospitalization and supportive care of some patients in the Colombia cohort reduced their risk for severe complications. Nevertheless, the highest-risk patients—those who progressed to SD despite supportive care—accordingly had the highest predicted probabilities of SD from the 8-gene model. Next, the class balance in the training data was inconsistent with real-world SD prevalence, leading our model to overestimate probabilities of SD in the Colombia cohort; however, calibration did not change any of our conclusions. Lastly, we measured gene expression in the Colombia cohort using a different platform (NanoString) than those used in the training datasets. Despite this, our model accurately distinguished SD progressors in the Colombia cohort, although the optimal (Youden) threshold differed from that in training. An important next step is to select a low-cost transcript measurement platform with rapid turnaround time for implementation of the 8-gene model, after which model recalibration and threshold selection can be performed. Several point-of-care platforms are now available that offer reliable measurement of RNA targets and subsequent application of machine learning algorithms to compute a risk score in as little as an hour [76, 77]. Such platforms could accommodate a prognostic assay based on the eight genes alone or in conjunction with DENV probes for rapid, simultaneous diagnosis of DENV infection and prediction of disease severity.

## Conclusions

The 8-gene XGBoost model, trained on heterogeneous public data, predicted progression to SD in a large independent cohort, improving upon clinical warning signs in accuracy, generalizability, and early prognostic power. Translated to a point-of-care prognostic assay, the 8-gene model has potential to improve dengue patient triage, guide treatment decisions, and reduce dengue morbidity and mortality without overwhelming healthcare resources.

## Supplementary Information

**Additional file 1.** Supplementary methods.

**Additional file 2: Table S1.** Description of 12 studies evaluated for benchmarking analysis. **Table S3.** Datasets included in the analysis of 8-gene signature performance in distinguishing severe outcomes for patients with other viral infections. **Table S4.** Description of the eight genes in the model and their mean effect size and heterogeneity (τ^2^ ) values across all 100 iterations. **Table S5.** Statistical power to detect a range of AUCs depending on proportion of SD cases (P), given the sample size of the independent Colombia cohort (N=377). **Table S6.** Age-specific performance of the 8-gene XGBoost model and clinical warning signs at presentation in the independent cohort.

**Additional file 3: Figure S1.** Diagram of patients excluded from the independent Colombia cohort. **Figure S2.** Linear classifiers are age-dependent in public datasets. **Figure S3.** 8-gene XGBoost model predicts progression to SD in public datasets. **Figure S4.** Performance of two previously published gene signatures for predicting SD progression. **Figure S5.** 8-gene XGBoost model predictions improve precision and are generalizable by age and clinical classification in the Colombia cohort. **Figure S6.** Calibration of 8-gene XGBoost model to proportion of SD cases observed in the Colombia cohort. **Figure S7.** Model predictions are associated with some clinical features in the Colombia cohort. **Figure S8.** 8-gene signature may generalize to other viral infections.

**Additional file 4: Table S2.** Clinical data, expression of genes in 8- and 20-gene signatures, and 8-gene XGBoost model predicted probabilities for patients in the Colombia cohort.

## Data Availability

All newly generated, de-identified data supporting the conclusions of this study are included within the article and its additional files.
