# Supplementary material for: An 8-gene machine learning model improves clinical prediction of severe dengue progression
Source: Genome Med. 2022 Mar 29;14:33. doi: 10.1186/s13073-022-01034-w (PMC8959795; doi:10.1186/s13073-022-01034-w)
Supplement: Supplementary file 1 — Additional file 1. Supplementary methods. [file 13073_2022_1034_MOESM1_ESM.docx]

**Additional File 1: Supplementary Methods**

**Pre-Processing of Public Dengue Datasets**

For one study, GSE43777, which contained samples from patients assayed on different microarray platforms (GPL201 and GPL570), we removed duplicate samples that were measured on both platforms such that each patient was only represented once in our analysis. We thereby considered GSE43777_GPL201 and GSE43777_GPL570 to be independent datasets.

We downloaded microarray datasets using getGEOData from the *MetaIntegrator* R package [1] and applied log2 transformation and quantile normalization when necessary. We used *kallisto* [2] for pseudoalignment of the RNA-seq dataset (GSE94892) to reference genome GRCh38 and *EdgeR* [3, 4] for normalization. Healthy controls and patients with other febrile illness were removed from all datasets prior to multi-cohort analysis. Longitudinal and convalescent samples were also removed, such that only one sample per patient, taken at the earliest timepoint, was included.

One study (GSE40628) specified the DHF grades of severity (DHFI-IV); for this dataset we classified patients with DHF grades III-IV as “SD progressors” and the rest as “non-severe,” based on the WHO 1997 definition of severe illness and a report in which the majority (86.5%) of DHFI-II cases were reclassified as D or DWS under the 2009 classification scheme [5, 6].

**Calculation of Standardized Expression Values for Longitudinal Analysis**

Each dataset had different proportions of cases and controls, which could influence standardization of expression values. We therefore sought to balance the classes in each dataset prior to standardization. Briefly, within each dataset, we calculated mean and standard deviation for each gene using all observations from the underrepresented class and an equivalent sample of observations from the overrepresented class, so that the two classes were equally represented. Then, we calculated standardized (centered and scaled) expression values for all observations using the intra-dataset mean and standard deviation. We then combined and analyzed standardized expression across all seven datasets over the disease course.

**Confirmation of Dengue Diagnosis in Colombia Cohort**

*rRT-PCR assays for detection of DENV and other microbial pathogens.* To confirm the diagnosis of dengue and differentiate from infection with the co-circulating arboviruses, Zika virus and chikungunya virus, serum samples were screened with a qualitative, single-reaction, multiplex real-time reverse transcriptase PCR (rRT-PCR) that detects Zika, chikungunya, and DENV RNA [7]. To identify the specific DENV serotype and determine the viral load, samples positive for DENV in the screening assay were serotyped and quantitated using a separate DENV multiplex rRT-PCR [8]. A single sample was also subjected to rRT-PCR for leptospira.

*DENV serological assays.* Anti-DENV IgG were tested using DENV Detect^TM^ IgG ELISA kit (InBios, Seattle, WA) as per manufacturer instructions. Briefly, serum samples were diluted 1:100 in sample dilution buffer and 50uL was added per well. The top half (4 wells on each 8-well strip) of the ELISA plate was pre-coated with DENV derived recombinant antigen (DENRA) and the other half with normal cell antigen (NCA). Each sample went into two pairs of DENRA or NCA coated wells, with or without 50 uL of 8 M Urea.

Each plate contained a negative and a positive control provided in the kit. Plates were incubated in 37 °C for 1 hour and washed in a plate washer (Biotek 404 Select Microwasher). Next, we added 50 uL/well enzyme conjugate-HRP tagged goat anti-human IgG and incubated for 1 hour at 37 °C, followed by wash. Next, 150 uL of EnWash was added, incubated for 5 min at room temperature, and washed. Finally, 75 uL of TMB solution was added and incubated at room temperature in the dark for 10 min. The reaction was stopped by adding 50 uL stop solution. The plates were read at 450 nM by Spectra Max M2 (Molecular Devices) using SoftMax pro 7.0.3 software.

Presence or absence of DENV IgG was interpreted from the ratio of readings from DENRA and NCA wells without urea. A ratio >=2.84 was considered positive, 1.65-2.84 ‘equivocal’ and <=1.65 negative. The equivocal samples were repeated once.

DENV IgG avidity was calculated by the ratio of readings in DENRA well without urea over the DENRA well with 8 M urea. High avidity (>0.6) was considered as secondary infection and <0.6 as primary infection, in samples that showed a positive result by DENRA/NCA ratio.

Multiplexed serological assays on a plasmonic-gold platform were used to measure DENV IgM and IgG as well as DENV IgG avidity in the first 36 patients enrolled, as described [9, 10].

1. Haynes, W.A., et al., *Empowering Multi-Cohort Gene Expression Analysis to Increase Reproducibility.* Pac Symp Biocomput, 2017. **22**: p. 144-153.

2. Bray, N.L., et al., *Near-optimal probabilistic RNA-seq quantification.* Nature Biotechnology, 2016. **34**(5): p. 525-527.

3. McCarthy, D.J., Y. Chen, and G.K. Smyth, *Differential expression analysis of multifactor RNA-Seq experiments with respect to biological variation.* Nucleic Acids Research, 2012. **40**(10): p. 4288-4297.

4. Robinson, M.D., D.J. McCarthy, and G.K. Smyth, *edgeR: a Bioconductor package for differential expression analysis of digital gene expression data.* Bioinformatics, 2010. **26**(1): p. 139-140.

5. Hadinegoro, S.R.S., *The revised WHO dengue case classification: does the system need to be modified?* Paediatrics and international child health, 2012. **32 Suppl 1**(s1): p. 33-38.

6. WHO, *Dengue haemorrhagic fever : diagnosis, treatment, prevention and control*. 1997, World Health Organization: Geneva.

7. Waggoner, J.J., et al., *Single-Reaction Multiplex Reverse Transcription PCR for Detection of Zika, Chikungunya, and Dengue Viruses.* Emerg Infect Dis, 2016. **22**(7): p. 1295-7.

8. Waggoner, J.J., et al., *Single-Reaction, Multiplex, Real-Time RT-PCR for the Detection, Quantitation, and Serotyping of Dengue Viruses.* PLoS Negl Trop Dis, 2013. **7**(4): p. e2116.

9. Zhang, B., et al., *Diagnosis of Zika virus infection on a nanotechnology platform.* Nat Med, 2017. **23**(5): p. 548-550.

10. Robinson, M., et al., *A 20-Gene Set Predictive of Progression to Severe Dengue.* Cell Rep, 2019. **26**(5): p. 1104-1111 e4.
