# Supplementary material for: An 8-gene machine learning model improves clinical prediction of severe dengue progression
Source: Genome Med. 2022 Mar 29;14:33. doi: 10.1186/s13073-022-01034-w (PMC8959795; doi:10.1186/s13073-022-01034-w)
Supplement: Supplementary file 2 — Additional file 2: Table S1. Description of 12 studies evaluated for benchmarking analysis. Table S3. Datasets included in the analysis of 8-gene signature performance in distinguishing severe outcomes for patients with other viral infections. Table S4. Description of the eight genes in the model and their mean effect size and heterogeneity (τ2 ) values across all 100 iterations. Table S5. Statistical power to detect a range of AUCs depending on proportion of SD cases (P), given the sample size of the independent Colombia cohort (N=377). Table S6. Age-specific performance of the 8-gene XGBoost model and clinical warning signs at presentation in the independent cohort. [file 13073_2022_1034_MOESM2_ESM.docx]

**Table S1: Description of 12 studies evaluated for benchmarking analysis.** GEO accession is listed for studies with associated public datasets. LDA, linear discriminant analysis; DEGs, differentially expressed genes.

| **Study** | **Description** |
| --- | --- |
| Nascimento *et al.* 2009 (GSE18090) [1] | 2-gene LDA-based model |
| Robinson *et al.* 2020 [2] | 20-gene geometric mean-based model |
| Nikolayeva *et al.* 2018 [3] | Excluded: 18-gene model, insufficient details to reproduce |
| Banerjee *et al.* 2017 (GSE94892) [4] | Excluded: 35 DEGs, no classification model |
| Hoang *et al.* 2010 (GSE25001) [5] | Excluded: 21 DEGs, no classification model |
| Long *et al.* 2009 (GSE13052) [6] | Excluded: 30 DEGs, no classification model |
| Sun *et al.* 2013 (GSE43777) [7] | Excluded: 7 DEGs, no classification model |
| Simmons *et al.* 2007 (GSE40628) [8] | Excluded: 22 DEGs, no classification model |
| Popper *et al*. 2012 (GSE38246) [9] | Excluded: 291 DEGs, not suitable for point-of-care test |
| Devignot *et al*. 2010 (GSE17924) [10] | Excluded: 2959 DEGs, not suitable for point-of-care test |
| Kwissa *et al*. 2014 (GSE51808) [11] | Excluded: no severity-associated DEGs |
| Simon-Loriere *et al*. 2017 (GSE100299) [12] | Excluded: no severity-associated DEGs |

**Table S3: Datasets included in the analysis of 8-gene signature performance in distinguishing severe outcomes for patients with other viral infections.** RSV, respiratory syncytial virus.

| **Dataset** | **Virus** | **Reference** | **Country** | **Age** | **Technology** | **N (non-severe, severe)** |
| --- | --- | --- | --- | --- | --- | --- |
| Inflammatix86 | SARS-CoV-2 | Giamarellos-Bourboulis *et al*. [13] | Greece | Adults | RNA sequencing | 62 (39, 23) |
| PRJNA390289 | Chikungunya | Michlmayr *et al.* [14] | Nicaragua | Children | RNA sequencing | 86 (44, 42) |
| GSE101702 | Influenza | Tang *et al.* [15]; Zerbib *et al.* [16] | Australia, Canada, Germany | Adults | Microarray | 107 (63, 44) |
| GSE77087 | RSV | de Steenhuijsen Piters *et al.* [17] | USA | Infants | Microarray | 81 (20, 61) |

**Table S4: Description of the eight genes in the model and their mean effect size and heterogeneity (τ^2^ ) values across all 100 iterations.** The summary effect size and τ^2^ (measure of heterogeneity) were computed for the training and validation cohorts in each iteration of multi-cohort analysis. The mean, standard deviation (StDev), and range across all 100 iterations are shown.

| **Gene** | **Gene Name** | **Description** | **Effect Size Mean (StDev)** | | **τ^2^  Mean [Range]** | |
| --- | --- | --- | --- | --- | --- | --- |
|  |  |  | **Training** | **Validation** | **Training** | **Validation** |
| *LTF* | Lactotransferrin | Nonspecific antiviral peptide secreted by neutrophils | 0.98 (0.20) | 0.96 (0.33) | 0.37 [0.12, 0.73] | 0.41 [0, 1.36] |
| *UQCRQ* | Ubiquinol-cytochrome c reductase complex III subunit VII | Subunit of mitochondrial electron transport chain complex III, part of oxidative phosphorylation | 0.57 (0.08) | 0.55 (0.16) | 0.03 [0, 0.18] | 0.06 [1.06e-5, 0.65] |
| *CKAP4* | Cytoskeleton associated protein 4 | ER membrane protein and cell surface receptor for various ligands including surfactant protein in the lung and DKK1 (Wnt inhibitor) | 0.50 (0.08) | 0.49 (0.14) | 0.01 [0, 0.05] | 0.02 [0, 0.19] |
| *ARNTL* | Aryl hydrocarbon receptor nuclear translocator like | Core circadian clock gene; may regulate the immune response in macrophages and neutrophils | -0.65 (0.13) | -0.67 (0.22) | 0.09 [0, 0.21] | 0.1 [0, 0.41] |
| *PDGFRB* | Platelet derived growth factor receptor beta | Cell surface tyrosine kinase receptor; essential for embryonic development and hematopoiesis | -0.64 (0.08) | -0.63 (0.16) | 0.02 [0, 0.06] | 0.04 [0, 0.28] |
| *TGFBR3* | Transforming growth factor beta receptor 3 | Transmembrane proteoglycan, binds TGF-beta isoforms and inhibins, involved in pathways regulating immune suppression and activation | -0.60 (0.09) | -0.60 (0.16) | 0.03 [0, 0.13] | 0.04 [0, 0.23] |
| *RASSF5* | Ras association domain family member 5 | Member of Ras effector super family protein involved in apoptosis, cell cycle regulation, differentiation | -0.45 (0.07) | -0.48 (0.14) | 0.02 [0, 0.08] | 0.03 [0, 0.22] |
| *GDPD5* | Glycerophosphodiester phosphodiesterase domain containing 5 | Transmembrane phosphodiesterase involved in glycerol metabolism | -0.57 (0.13) | -0.60 (0.22) | 0.07 [0, 0.19] | 0.11 [0, 0.2] |

**Table S5: Statistical power to detect a range of AUCs depending on proportion of SD cases (P), given the sample size of the independent Colombia cohort (N=377).**

| **AUROC** | **P = 0.02** | **P = 0.04** | **P = 0.058** | **P = 0.08** | **P = 0.10** | **P = 0.20** |
| --- | --- | --- | --- | --- | --- | --- |
| 0.60 | 0.17 | 0.27 | 0.37 | 0.46 | 0.55 | 0.79 |
| 0.65 | 0.32 | 0.52 | 0.68 | 0.80 | 0.88 | 0.99 |
| 0.70 | 0.51 | 0.77 | 0.90 | 0.96 | 0.99 | 1.00 |
| 0.75 | 0.70 | 0.92 | 0.98 | 1.00 | 1.00 | 1.00 |
| 0.80 | 0.86 | 0.99 | 1.00 | 1.00 | 1.00 | 1.00 |
| 0.85 | 0.96 | 1.00 | 1.00 | 1.00 | 1.00 | 1.00 |
| 0.90 | 1.00 | 1.00 | 1.00 | 1.00 | 1.00 | 1.00 |
| 0.95 | 1.00 | 1.00 | 1.00 | 1.00 | 1.00 | 1.00 |

**Table S6: Age-specific performance of the 8-gene XGBoost model and clinical warning signs at presentation in the independent cohort.** 95% confidence intervals (CIs) from bootstrapping are shown in parentheses for each metric. SD, severe dengue; PPV, positive predictive value; NPV, negative predictive value.

|  |  | **Sensitivity %** | **Specificity %** | **PPV %** | **NPV %** |
| --- | --- | --- | --- | --- | --- |
| **Children**  **(4% SD)** | **8-gene XGBoost Model** | 90.0 (66.7-100.0) | 85.0 (80.3-89.4) | 20.0 (10.6-30.6) | 99.5 (98.5-100.0) |
|  | **Warning Signs** | 90.0 (66.7-100.0) | 37.1 (31.0-43.3) | 5.6 (3.0-8.4) | 98.9 (96.4-100.0) |
| **Adults**  **(9.4% SD)** | **8-gene XGBoost Model** | 83.3 (60.0-100.0) | 68.7 (59.8-77.3) | 21.7 (12.5-31.9) | 97.5 (93.9-100.0) |
|  | **Warning Signs** | 66.7 (38.5-91.7) | 45.2 (36.0-54.3) | 11.3 (5.5-17.4) | 92.9 (86.3-98.3) |

1. Nascimento EJ, Braga-Neto U, Calzavara-Silva CE, Gomes AL, Abath FG, Brito CA, et al. Gene expression profiling during early acute febrile stage of dengue infection can predict the disease outcome. PLoS One. 2009;4(11):e7892. Epub 2009/11/26. doi: 10.1371/journal.pone.0007892. PubMed PMID: 19936257; PubMed Central PMCID: PMCPMC2775946.

2. Robinson M, Sweeney TE, Barouch-Bentov R, Sahoo MK, Kalesinskas L, Vallania F, et al. A 20-Gene Set Predictive of Progression to Severe Dengue. Cell Rep. 2019;26(5):1104-11 e4. Epub 2019/01/31. doi: 10.1016/j.celrep.2019.01.033. PubMed PMID: 30699342; PubMed Central PMCID: PMCPMC6352713.

3. Nikolayeva I, Bost P, Casademont I, Duong V, Koeth F, Prot M, et al. A Blood RNA Signature Detecting Severe Disease in Young Dengue Patients at Hospital Arrival. The Journal of Infectious Diseases. 2018;217(11):1690-8. doi: 10.1093/infdis/jiy086.

4. Banerjee A, Shukla S, Pandey AD, Goswami S, Bandyopadhyay B, Ramachandran V, et al. RNA-Seq analysis of peripheral blood mononuclear cells reveals unique transcriptional signatures associated with disease progression in dengue patients. Transl Res. 2017;186:62-78 e9. Epub 2017/07/07. doi: 10.1016/j.trsl.2017.06.007. PubMed PMID: 28683259.

5. Hoang LT, Lynn DJ, Henn M, Birren BW, Lennon NJ, Le PT, et al. The early whole-blood transcriptional signature of dengue virus and features associated with progression to dengue shock syndrome in Vietnamese children and young adults. J Virol. 2010;84(24):12982-94. Epub 2010/10/15. doi: 10.1128/JVI.01224-10. PubMed PMID: 20943967; PubMed Central PMCID: PMCPMC3004338.

6. Long HT, Hibberd ML, Hien TT, Dung NM, Van Ngoc T, Farrar J, et al. Patterns of gene transcript abundance in the blood of children with severe or uncomplicated dengue highlight differences in disease evolution and host response to dengue virus infection. J Infect Dis. 2009;199(4):537-46. Epub 2009/01/14. doi: 10.1086/596507. PubMed PMID: 19138155; PubMed Central PMCID: PMCPMC4333209.

7. Sun P, Garcia J, Comach G, Vahey MT, Wang Z, Forshey BM, et al. Sequential waves of gene expression in patients with clinically defined dengue illnesses reveal subtle disease phases and predict disease severity. PLoS Negl Trop Dis. 2013;7(7):e2298. Epub 2013/07/23. doi: 10.1371/journal.pntd.0002298. PubMed PMID: 23875036; PubMed Central PMCID: PMCPMC3708824.

8. Simmons CP, Popper S, Dolocek C, Chau TN, Griffiths M, Dung NT, et al. Patterns of host genome-wide gene transcript abundance in the peripheral blood of patients with acute dengue hemorrhagic fever. J Infect Dis. 2007;195(8):1097-107. Epub 2007/03/16. doi: 10.1086/512162. PubMed PMID: 17357045; PubMed Central PMCID: PMCPMC4042601.

9. Popper SJ, Gordon A, Liu M, Balmaseda A, Harris E, Relman DA. Temporal dynamics of the transcriptional response to dengue virus infection in Nicaraguan children. PLoS Negl Trop Dis. 2012;6(12):e1966. Epub 2013/01/04. doi: 10.1371/journal.pntd.0001966. PubMed PMID: 23285306; PubMed Central PMCID: PMCPMC3527342.

10. Devignot S, Sapet C, Duong V, Bergon A, Rihet P, Ong S, et al. Genome-wide expression profiling deciphers host responses altered during dengue shock syndrome and reveals the role of innate immunity in severe dengue. PLoS One. 2010;5(7):e11671. Epub 2010/07/24. doi: 10.1371/journal.pone.0011671. PubMed PMID: 20652028; PubMed Central PMCID: PMCPMC2907396.

11. Kwissa M, Nakaya HI, Onlamoon N, Wrammert J, Villinger F, Perng GC, et al. Dengue virus infection induces expansion of a CD14(+)CD16(+) monocyte population that stimulates plasmablast differentiation. Cell Host Microbe. 2014;16(1):115-27. Epub 2014/07/02. doi: 10.1016/j.chom.2014.06.001. PubMed PMID: 24981333; PubMed Central PMCID: PMCPMC4116428.

12. Simon-Loriere E, Duong V, Tawfik A, Ung S, Ly S, Casademont I, et al. Increased adaptive immune responses and proper feedback regulation protect against clinical dengue. Sci Transl Med. 2017;9(405). Epub 2017/09/01. doi: 10.1126/scitranslmed.aal5088. PubMed PMID: 28855396.

13. Giamarellos-Bourboulis EJ, Netea MG, Rovina N, Akinosoglou K, Antoniadou A, Antonakos N, et al. Complex Immune Dysregulation in COVID-19 Patients with Severe Respiratory Failure. Cell Host Microbe. 2020;27(6):992-1000.e3. Epub 2020/04/23. doi: 10.1016/j.chom.2020.04.009. PubMed PMID: 32320677; PubMed Central PMCID: PMCPMC7172841.

14. Michlmayr D, Pak TR, Rahman AH, Amir ED, Kim EY, Kim-Schulze S, et al. Comprehensive innate immune profiling of chikungunya virus infection in pediatric cases. Mol Syst Biol. 2018;14(8):e7862. Epub 2018/08/29. doi: 10.15252/msb.20177862. PubMed PMID: 30150281; PubMed Central PMCID: PMCPMC6110311.

15. Tang BM, Shojaei M, Teoh S, Meyers A, Ho J, Ball TB, et al. Neutrophils-related host factors associated with severe disease and fatality in patients with influenza infection. Nat Commun. 2019;10(1):3422. Epub 2019/08/02. doi: 10.1038/s41467-019-11249-y. PubMed PMID: 31366921; PubMed Central PMCID: PMCPMC6668409.

16. Zerbib Y, Jenkins EK, Shojaei M, Meyers AFA, Ho J, Ball TB, et al. Pathway mapping of leukocyte transcriptome in influenza patients reveals distinct pathogenic mechanisms associated with progression to severe infection. BMC Med Genomics. 2020;13(1):28. Epub 2020/02/19. doi: 10.1186/s12920-020-0672-7. PubMed PMID: 32066441; PubMed Central PMCID: PMCPMC7027223.

17. de Steenhuijsen Piters WA, Heinonen S, Hasrat R, Bunsow E, Smith B, Suarez-Arrabal MC, et al. Nasopharyngeal Microbiota, Host Transcriptome, and Disease Severity in Children with Respiratory Syncytial Virus Infection. Am J Respir Crit Care Med. 2016;194(9):1104-15. Epub 2016/11/01. doi: 10.1164/rccm.201602-0220OC. PubMed PMID: 27135599; PubMed Central PMCID: PMCPMC5114450.
