# Supplementary material for: An 8-gene machine learning model improves clinical prediction of severe dengue progression
Source: Genome Med. 2022 Mar 29;14:33. doi: 10.1186/s13073-022-01034-w (PMC8959795; doi:10.1186/s13073-022-01034-w)
Supplement: Supplementary file 3 — Additional file 3: Figure S1. Diagram of patients excluded from the independent Colombia cohort. Figure S2. Linear classifiers are age-dependent in public datasets. Figure S3. 8-gene XGBoost model predicts progression to SD in public datasets. Figure S4. Performance of two previously published gene signatures for predicting SD progression. Figure S5. 8-gene XGBoost model predictions improve precision and are generalizable by age and clinical classification in the Colombia cohort. Figure S6. Calibration of 8-gene XGBoost model to proportion of SD cases observed in the Colombia cohort. Figure S7. Model predictions are associated with some clinical features in the Colombia cohort. Figure S8. 8-gene signature may generalize to other viral infections. [file 13073_2022_1034_MOESM3_ESM.docx]

**Fig S1: Diagram of patients excluded from the independent Colombia cohort.**

**Fig S2: Linear classifiers are age-dependent in public datasets.** Violin plots of **A)** geometric mean classifier and **B)** logistic regression classifier for public datasets profiling children, adults, or a combination of children and adults. ROC curves of **C)** geometric mean classifier and **D)** logistic regression classifier in distinguishing non-severe patients from SD progressors in public datasets that profiled children (red), adults (blue), or both children and adults (orange). The DeLong test p-value=0.071 for the geometric mean classifier and 0.029 for the logistic regression classifier when comparing ROCs for children vs. adults.

**Fig S3: 8-gene XGBoost model predicts progression to SD in public datasets. A)** Violin plots of 8-gene model predictions in each of the nine public datasets used for model training. **B)** ROC curves of the 8-gene model in distinguishing non-severe dengue patients and SD progressors in the public datasets (violin plots and ROC curves not shown for two datasets that did not measure all eight genes). The thick black line and grey shaded area represent the smoothed summary ROC curve and its weighted standard deviation, respectively (Methods). **C)** Violin plots of predicted probabilities of progression to SD in public datasets profiling children only, adults only, or children and adults. The dotted horizontal line indicates the Youden optimal threshold, computed over all public datasets combined.

**Fig S4: Performance of two previously published gene signatures for predicting SD progression. A)** Performance of 2-gene classifier described by Nascimento *et al.* in other public datasets. The published model uses linear discriminant analysis to separate uncomplicated dengue fever (DF) patients from dengue hemorrhagic fever (DHF) patients, such that “lower expression of both genes is a signature for DHF, whereas higher expression of both genes is a signature for DF.” **B)** Performance of 20-gene signature described by Robinson *et al.* in public datasets not used for training.

**Fig S5: 8-gene XGBoost model predictions improve precision and are generalizable by age and clinical classification in the Colombia cohort. A)** Precision-recall curve for the 8-gene XGBoost model in the independent Colombia cohort with 5.8% SD cases. The black point indicates the precision and recall of the 8-gene model at the Youden threshold; the red point indicates the precision and recall of clinical warning signs. AUPRC, area under the precision-recall curve. **B)** ROC curves depicting performance of the prior 20-gene set (dashed lines) versus the 8-gene XGBoost model (solid lines) in predicting progression to SD among adults (blue) or children (red) in the Colombia cohort. The DeLong p-value (adults versus children) is 0.0026 for the 20-gene set and 0.19 for the 8-gene set. **C)** 8-gene model predictions on patients reclassified using the 1997 WHO criteria. According to the 1997 criteria, uncomplicated dengue and dengue hemorrhagic fever grades 1-2 (DHFI-II) are considered non-severe, and grade 3 (DHFIII) and DSS are considered severe. Organ damage is included as a manifestation of SD in the WHO 2009 criteria but not the 1997 criteria. The dotted horizontal line indicates the Youden threshold calculated using the 2009 criteria. **D)** ROC curve of the 8-gene XGBoost model in predicting severe disease using the 1997 WHO classification criteria.

**Fig S6: Calibration of 8-gene XGBoost model to proportion of SD cases observed in the Colombia cohort. A)** Uncalibrated and calibrated mean of predicted probabilities from the 8-gene model within each of seven bins compared to observed probability of SD progression among patients in the corresponding bin. **B)** Calibrated predicted probabilities in the training data (where controls were resampled to match the proportion of SD cases observed in the test data; left), and in the test data (Colombia cohort; right). Dotted horizontal lines indicate the Youden thresholds following calibration.

**Fig S7:** **Model predictions are associated with some clinical features in the Colombia cohort.** Scatter and violin plots depicting the relationships between the 8-gene model predictions and a variety of relevant clinical warning signs and parameters: **A)** prior exposure to DENV measured by IgG avidity, **B)** vomiting, **C)** hemorrhage, **D)** abdominal pain, **E)** fluid accumulation, **F)** hepatomegaly, **G)** peak aspartate transaminase (AST), **H)** peak alanine transaminase (ALT), **I)** platelet nadir. For continuous parameters, *R* and *p* correspond to Pearson correlation coefficients and significance test p-values, respectively. For discrete variables, Wilcoxon signed rank test p values are shown.

**Fig S8: 8-gene signature may generalize to other viral infections.** Performance of 8-gene signature in distinguishing severe and non-severe patients with SARS-CoV-2, chikungunya, influenza, or respiratory syncytial virus (RSV) infection.
